# Modified serpinA1 as risk marker for Parkinson’s disease dementia: Analysis of baseline data

**DOI:** 10.1038/srep26145

**Published:** 2016-05-17

**Authors:** Steffen Halbgebauer, Magdalena Nagl, Hans Klafki, Ute Haußmann, Petra Steinacker, Patrick Oeckl, Jan Kassubek, Elmar Pinkhardt, Albert C. Ludolph, Hilkka Soininen, Sanna-Kaisa Herukka, Jens Wiltfang, Markus Otto

**Affiliations:** 1 Department of Neurology, University of Ulm, Germany; 2 LVR-Klinikum Essen, Department of Psychiatry and Psychotherapy, Faculty of Medicine, University of Duisburg-Essen, Germany; 3 Department of Psychiatry and Psychotherapy, University Medical Center (UMG), Georg-August-University, Göttingen, Germany; 4 Department of Neurology, University of Eastern Finland and Kuopio University Hospital, Kuopio, Finland

## Abstract

Early detection of dementia in Parkinson disease is a prerequisite for preventive therapeutic approaches. Modified serpinA1 in cerebrospinal fluid (CSF) was suggested as an early biomarker for differentiation between Parkinson patients with (PDD) or without dementia (PD). Within this study we aimed to further explore the diagnostic value of serpinA1. We applied a newly developed nanoscale method for the detection of serpinA1 based on automated capillary isoelectric focusing (CIEF). A clinical sample of 102 subjects including neurologically healthy controls (CON), PD and PDD patients was investigated. Seven serpinA1 isoforms of different charge were detected in CSF from all three diagnostic groups. The mean CSF signals of the most acidic serpinA1 isoform differed significantly (p < 0.01) between PDD (n = 29) and PD (n = 37) or CON (n = 36). Patients above the cut-off of 6.4 have a more than six times higher risk for an association with dementia compared to patients below the cut off. We propose this serpinA1 CIEF-immunoassay as a novel tool in predicting cognitive impairment in PD patients and therefore for patient stratification in therapeutic trials.

Parkinson’s disease (PD) is one of the most common neurodegenerative disorders^1^. Besides the cardinal symptoms of PD, symptoms of mild cognitive impairment can be seen in up to 50% of PD patients^2^,^3^. Particularly these non-motor symptoms have a high impact on the quality of life of PD patients and often lead to an earlier hospitalisation^4^. PD patients have a 6 times higher risk to develop a dementia than the general population at the same age^5^. The dementia syndrome accelerates disease progression and thereby decreases the life expectancy of the Parkinson’s disease with dementia (PDD) patients^6^. There are still no robust neurochemical biomarkers available which support the clinical diagnosis of dementia in PD patients.

In 2012, our group described a new promising marker for the prediction of cognitive impairment in PD patients^7^. When analysed by isoelectric focusing and subsequent SDS-polyacrylamide electrophoresis (2D-PAGE), CSF of PDD patients showed differently sialylated serpinA1 isoforms compared to controls and PD patients. However, 2D-PAGE is time-consuming and cannot be used as a high-throughput approach.

SerpinA1 is an acute phase protein, belonging to the serpin superfamily^8^,^9^. It is a plasma serine protease inhibitor. It is assumed that by diffusion out of venous blood, the plasma protein serpinA1 also ends up in the CSF. In addition, serpinA1 was reported to be released from the brain tissue into the CSF^7^.

Here we report on the development of a new capillary isoelectric focusing immunoassay (CIEF-immunoassay) for the analysis of serpinA1 charge isoforms in CSF and its ability to differentiate between cognitive normal and demented Parkinson patients. Therefore, we applied cross-sectional investigations on a clinical cohort of 102 longitudinally followed subjects in total.

## Results

### CSF serpinA1 analysis by 2D-PAGE

To confirm the previous findings of differently sialylated isoforms of serpinA1 in the CSF of control, PD and PDD patients (Jesse *et al*.^7^), we performed two-dimensional (2D) serpinA1 immunoblots of CSF samples from 12 controls, 13 PD and 12 PDD patients.

All of the tested control samples and 12 out of 13 tested PD CSF samples showed a similar pattern of five serpinA1 spots slightly above the 50 kDa marker protein band. The one remaining PD immunoblot showed an additional sixth spot. The 2D immunoblots of CSF from PDD patients showed different patterns of serpinA1 charge isoforms than controls and PDs: 10 out of 12 immunoblots showed six spots whereas only two immunoblots showed less than six spots (See Supplementary Fig. S1).

The detection of more than five charge isoforms of serpinA1 in PDD patients compared to PD patients and controls confirms serpinA1 to represent a potential marker for the diagnosis of dementia in PD patients.

### CSF serpinA1 analysis by CIEF-immunoassay

**Figure 1:**
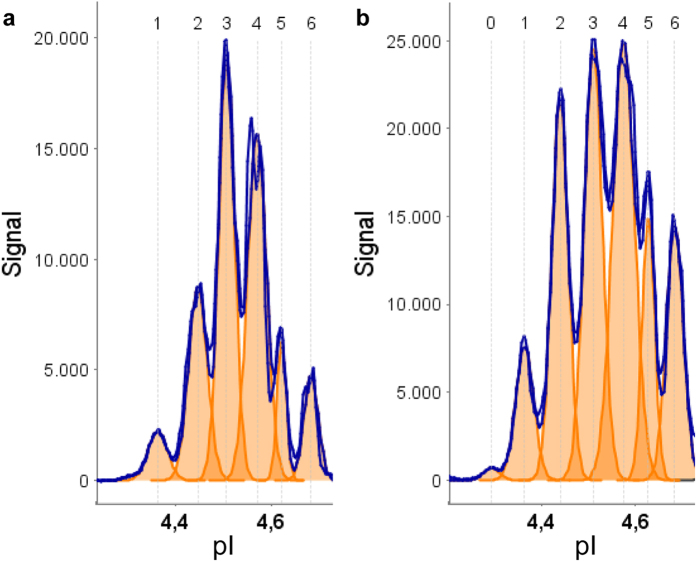
Two typical CSF serpinA1 electropherograms. (**a**) Electropherogram of a control CSF showing six distinct peaks around the pI of 4.5. (**b**) electropherogram of a PDD CSF showing seven distinct peaks. The additional peak 0 was always found on acidic side of the other peaks. The orange color indicates the single peak areas. Signal intensity in chemiluminescence units; pI, isoelectric point. Exposure time 30 s.

**Figure 2:**
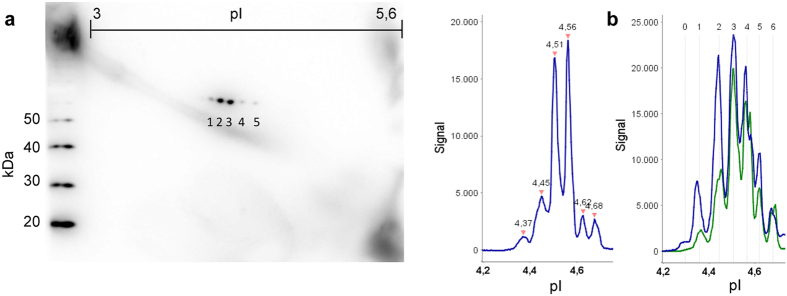
Detection of serpinA1 charge isoforms by 2D immunoblot and CIEF immunoassay. (**a**) Comparison of a control 2D serpinA1 immunoblot with the corresponding CIEF electropherogram. The peaks at 4.51 and 4.56 match the two intense spots from the 2D immunoblot. The two more basic and one more acidic isoform can also be found in the immunoblot. However, the most acidic peak in the electropherogram cannot be seen in the 2D immunoblot. (**b**) Overlay of one control (green) and one PDD (blue) electropherogram. The difference in the relative abundances especially of the more acidic isoforms can clearly be seen. The isoforms corresponding to peak 1 and peak 2 are noticeably increased in the PDD sample as compared to controls. Peak 0 is only detectable in the PDD patient. Signal intensity in chemiluminescence units; pI, isoelectric point. 2D exposure time 2 s. CIEF exposure time 30 s.

To evaluate the feasibility of capillary isoelectric focusing for the measurement of serpinA1 in human CSF we analysed 102 CSF samples in total, including samples from n = 36 controls, n = 37 PD and n = 29 PDD. In Fig. 1A, a typical CIEF electropherogram of a control sample is shown as an example. In all 102 samples analysed, at least six distinct serpinA1 peaks were observed with isoelectric points ranging from pI 4.3 to pI 4.7 (peaks 1–6). However, in a number of samples even a seventh peak on the acidic side was found (peak 0) (Fig. 1B). In nearly all electropherograms the two most intense peaks had pIs between pH 4.5 and 4.6. These two serpinA1 isoforms were always accompanied by less abundant isoforms on their acidic and basic sides. This is similar to the observations from 2D immunoblots on which also at least two comparatively intense spots were detected, that were accompanied by additional, weaker protein spots. Because of this similarity these two intense spots and peaks were matched as corresponding isoforms (Fig. 2A).

Evaluating the CIEF electropherograms of the three different groups we saw differences in the abundance of certain serpinA1 isoforms. As an example, in Fig. 2B an overlay of two electropherograms of one control and one PDD sample is shown. It appears that the PDD sample shows a substantial relative increase of the acidic serpinA1 isoforms (peaks 0, 1 and 2) as compared to the control.

### Analysis of peak areas

**Figure 3:**
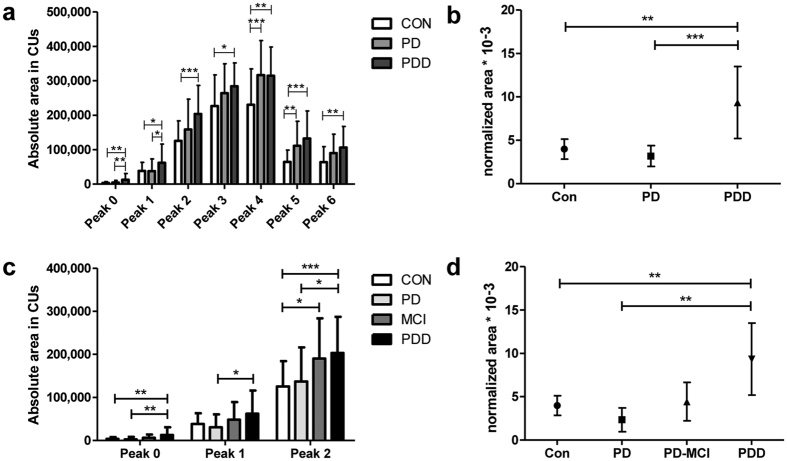
Results of peak area analysis. (**a**) Absolute mean peak areas of peaks 0–6. 36 controls, 37 PDs and 29 PDDs were analysed. Error bars indicate SD. (**b**) Normalized peak areas of peak 0 relative to the summed up area of all peaks are indicated. Significant group differences between PDD and control/PD patients. CON = 4.0 (95% CI 2.8 to 5.1), PDs = 3.2 (95% CI 2.0 to 4.4) and PDDs = 9.3 (95% CI 5.2 to 13.5) (normalized peak area * 10^−3^). Symbols indicate mean normalized area. Error bars indicate 95% confidence interval. (**c**) Absolute mean peak areas of peaks 0–2 after grouping of the PD patients into PD witch normal cognitive function and mild cognitive impairment. (**d**) Normalized peak area of peak 0 after stratification of the PD patients into PD with normal cognitive function and PD-MCI. CON, control; PD, Parkinson’s disease; PD-MCI, Parkinson’s disease with mild cognitive impairment; PDD, Parkinson’s disease with dementia; *p < 0.05 **p < 0.01; ***p < 0.001.

For a semi-quantitative comparison of the serpinA1 levels and isoform patterns in the three groups, peak area calculations were performed. Signal intensities were calculated for each serpinA1 isoform individually and for total serpinA1 as the sum of all individual peaks. The mean overall serpinA1 CSF signals were statistically significantly higher in PDD samples than in controls (p < 0.001) and also in PD than in controls (p < 0.05) (See Supplementary Fig. S2). Furthermore, significant group differences between PDD and control samples were found for each individual serpinA1 peak. Statistically significant group differences between PD and PDD were observed only for the two most acidic serpinA1 peaks, peak 0 (p < 0.01) and peak 1 (p < 0.05), but not for total serpinA1 signal or any of the remaining serpinA1 charge isoforms (Fig. 3A).

### Receiver Operating Characteristic analysis of Peak 0

**Figure 4:**
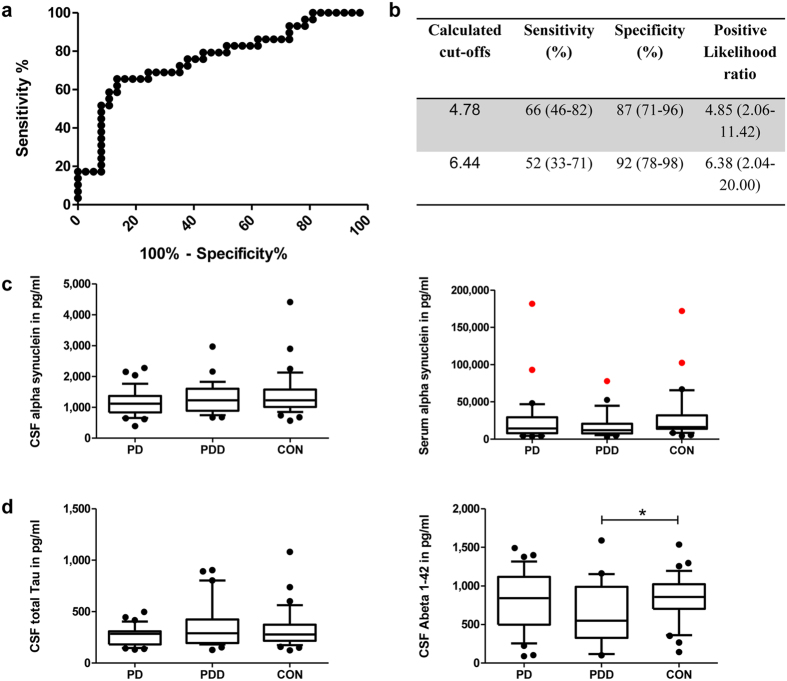
ROC of normalized peak 0 and display of alpha-synuclein, tau and Abeta 1–42 levels. (**a**) Receiver operating characteristics (ROC) curve of normalized peak 0 from PD vs. PDD. 37 PD and 29 PDD patients were analysed. Area under the curve: 0.76. (**b**) Calculated cut-offs with corresponding sensitivity, specificity and positive likelihood ratio for the normalized area of peak 0 from PD and PDD. 95% confidence interval in brackets. (**c**) Display of alpha-synuclein levels in CSF (left) and serum (right) of PD, PDD and control samples. Red colour indicates serum samples that were contaminated with erythrocytes and were thus considered outliers. These samples were excluded from further analyses. No significant differences between the three groups were found. (**d**) Display of total Tau levels in CSF (left) and Abeta 1–42 (right) of PD, PDD and control samples. Only the Abeta 1–42 levels of control and PDD samples showed a statistically significant difference . In the box plots the median concentrations are shown, 25% and 75% percentile, and 10% and 90% whiskers. CON, control; PD, Parkinson’s disease; PDD, Parkinson’s disease with dementia.

To evaluate the diagnostic potential of serpinA1 peak 0 for the discrimination between PD and PDD, we first normalized peak 0 to the summed up peak areas (total serpinA1 signal) (Fig. 3B) and then performed Receiver Operating Characteristic (ROC) analysis (Fig. 4A). The cut-off value regarding sensitivity and specificity was calculated with Youden’s index (1^st^ cut off). In addition a cut-off with a higher selectivity (value with highest likelihood ratio = 2^nd^ cut off) was selected. PD patients who have a higher normalized serpinA1 signal of the most acidic isoform (peak 0) than the selected 2^nd^ cut-off value of 6.44 * 10^−3^ have a 6.4 fold higher risk to be associated with dementia compared to PD patients with a normalized serpinA1 signal below this cut-off (Fig. 4B).

To analyse if serpinA1 could serve as a predictive biomarker for dementia in PD patients we stratified our PD patient cohort into PD with intact cognitive function (PD) and PD with mild cognitive impairment (PD-MCI) and analysed selected acidic peaks (Fig. 3C), especially the normalized peak 0 (Fig. 3D). The significant differences between control/PD and PDD patients could still be seen. Furthermore, PD-MCI patients showed an elevated serpinA1 level compared to PD patients but a lower one compared to PDD patients.

To exclude any influence of age on the serpinA1 isoform levels the pearson correlation coefficient between age and the absolute area of Peak 0 was determined. No significant correlation could be detected (r = 0.1275 and p = 0.2014) (See Supplementary Fig. S3). Furthermore, and to exclude age as confounding factor of serpinA1 isoform levels, we analyzed an additional control cohort of 20 patients with a mean age comparable to the PDD cohort. The mean absolute are of the most acidic isoform was comparable to the area observed in the control cohort of 36 patients (See Supplementary Fig. S4). Moreover, we checked the pearson correlation between the absolute area of peak 0 and the disease duration of the patients. Here, we could detect a weak correlation (r = 0.3366 and p = 0.0193). Performing a multivariate regression with disease duration as covariate the significant difference in absolute peak area 0 levels between PD and PDD maintained (data not shown).

### Comparison with alpha-synuclein, tau and Abeta 1–42

We measured the alpha-synuclein levels in the CSF and sera of our control, PD and PDD samples with the goal to compare them with the CIEF serpinA1 findings. CSF and serum alpha-synuclein levels as well as the quotient of both showed no significant differences between the three groups (See Fig. 4C and Supplementary Fig. S3). In addition, neither Tau nor Abeta 1–42 showed significant differences between all 3 groups (Fig. 4D).

## Discussion

The diagnosis of PDD is done according to clinical criteria^10^. Biochemical markers, however, could be highly valuable in predicting the conversion to dementia in patients with normal cognitive function and PD/PD-MCI patients. Thus, they may support an early initiation of appropriate therapeutic treatment.

Using CIEF we could confirm the findings by Jesse and colleagues that there are differences in the sialylation of serpinA1 isoforms in the CSF of control, PD and PDD patients. Especially the most acidic serpinA1 isoform (peak 0) discriminated the 37 measured PD from 29 PDD CSF samples (p < 0.01). Using a specific cut-off calculated with ROC analysis and Youden’s index we found a sensitivity of 66% and a specificity of 87%. In addition to this calculated cut-off we selected a 2^nd^ cut-off with a higher specificity. Our results indicate that PD patients in whom the level of the most acidic serpinA1 isoform is higher than this 2nd cut-off value have a 6.4 fold increased chance to be associated with dementia. This correlates with the finding that PD patients have a roughly six times higher chance to develop a dementia than healthy people at the same age^5^.

We speculate that the CSF serpinA1 isoform analysis by the novel CIEF-immunoassay may turn out to have value for the biomarker supported prediction of cognitive impairment, as the serpinA1 isoform pattern might already be altered at a pre-dementia stage in PD patients who will develop PDD. This hypothesis is supported by the finding that PD patients suffering from mild cognitive impairment showed elevated serpinA1 peak 0 levels compared to PD patients without MCI. It was reported that 36–55% of PD patients already suffer from cognitive impairment at the time of diagnosis^11^,^12^. As all our PD patients undergo a clinical follow up to be able to redefine the diagnosis longitudinally, the CSF serpinA1 levels of patients developing a cognitive decline over the years can be checked immediately.

So far, as we only can present baseline data, a limitation of the work is certainly its cross-sectional nature. Another important point is the weak correlation of serpinA1 peak 0 levels with disease duration, which we probably detect as a consequence of the significant differences in disease duration between the PDD and PD group (see Supplementary Table S2). This weak correlation could implicate that serpinA1 could also be a progression marker of the underlying pathology. We, however, believe that the correlation is due to the fact that a Parkinson associated dementia usually arises in the later disease course, whereas a mild cognitive impairment is often detected even in newly diagnosed patients^13^. In consideration that a Parkinson disease dementia occurs on average ten years after the first extrapyramidal signs^14^ and PDD is associated with a higher serpinA1 level of the most acidic isoform, it is not surprising that we can evaluate a correlation between disease duration and the serpinA1 level of peak 0. In addition, for future analyses a tighter pH gradient e.g. from pH 3–5 for CIEF may help to further improve the detection of serpinA1 isoforms by increasing the resolution in the pH area of interest.

For comparison of our serpinA1 findings with a frequently analysed biomarker candidate in PD we chose to analyse alpha-synuclein levels in CSF and serum of our patients. In the literature alpha-synuclein is not^15^,^16^,^17^ or only slightly decreased^18^,^19^,^20^,^21^ in the CSF of PD compared to control patients. We could also find no significant differences in CSF or serum alpha-synuclein levels (only a small decrease of alpha-synuclein in PD patients) between the three groups emphasizing the potential diagnostic value of CIEF serpinA1 measurements for the discrimination between control, PD and PDD patients.

Little is known about the possible pathophysiological role of post-translational modified serpinA1 in the development of PDD. In general, it has been shown that serpinA1 is able to polymerize and form aggregates^22^,^23^. Such pathological accumulation of serpin polymers has been linked to several human diseases for example in forms of liver cirrhosis^24^. SerpinA1 is closely homologous to neuroserpin^25^ another serpin family member and it is very interesting that the histochemistry of serpinA1 aggregates is very similar to inclusions found in neurodegenerative disease caused by intracellular accumulation of mutant neuroserpin; familial encephalopathy with neuroserpin inclusion bodies^26^. In addition serpinA1 levels seem to be increased in the CSF of PDD and DLB patients^7^,^27^.

Phosphorylation events during neurodegeneration are widely accepted. In this context Wang *et al*. described that phosphorylated alpha-synuclein can discriminate between PD and atypical parkinsonism, showing that post-translational modifications are indeed a tool to distinguish diseases^28^. Protein glycosylation has also been described to be involved in the pathogenesis of neurodegenerative diseases^29^,^30^,^31^. However, the biochemical mechanism of sialylation in neurodegenerative diseases is less explored. Jesse *et al*. demonstrated that in serpinA1 an O-linked sialylation is present^7^. The sialylation event in serpinA1 might therefore be in direct competition with phosphorylation as they share similar motifs. On one hand, sialylation of serpinA1 might serve to protect from hyperphosphorylation. That sialylation in principle can suppress phosphorylation has been demonstrated^32^,^33^. On the other hand hypersialylation might lead to an aberrant structure promoting aggregation in the course of the disease. To confirm or dismiss these speculations further studies in suitable experimental models will have to be performed.

To conclude, we demonstrate that the novel serpinA1 CIEF-immunoassay can help to discriminate PDD patients from control and PD patients in a standardized, fast and high-throughput compatible way. As criteria for discrimination the relative levels of certain serpinA1 isoforms represent the decisive factor. Moreover, the CSF serpinA1 isoform analysis might already predict cognitive impairment in PD patients who will develop a dementia in the course of the disease. We found that patients with a positive serpinA1 test have a more than 6 times higher risk of an association with dementia. The measurement of serpinA1 can therefore support the early clinical diagnosis of dementia in PD patients and help to stratify patient populations for therapeutic trials.

## Material and Methods

### Patients

Subjects were recruited through the Department of Neurology of the “Universitäts- und Rehabilitationskliniken Ulm (RKU)” and the Department of Neurology, Kuopio, Finland, in the time period of April 2012 to August 2014 as part of a prospective study to find biomarkers for PD and PDD (Valid-PDD). Baseline data of these patients are presented. The study was approved by the Ethics Committee of the University of Ulm approval numbers: 8801 and 100305 as well as the Ethics Committee of Kuopio University Hospital (number 5/2002). All methods were carried out in accordance with the approved guidelines. All patients signed an informed consent previous to study enrolment.

A total of 66 Parkinson patients, thereof n = 22 without (PD), n = 15 with mild cognitive impairment (PD-MCI) and 29 patients with Parkinson disease dementia (PDD) as well as 36 non demented controls (CON) were examined.

All participants underwent extensive evaluation consisting of physical and neurological examination, a 1.5 or 3 Tesla brain magnetic resonance imaging (MRI), blood screening and lumbar puncture. All patients enrolled received their usual dopaminergic medication. Current and history pharmacological treatment were recorded at the time of assessment to ensure that the therapy had been stable for at least 3 months (for detailed information about the given antiparkinson medication see Supplementary Table S1). To rule out possible concomitant etiological or at least aggravating factors of the present dementia symptoms, we excluded all patients with current or history of other neurological diseases. PD was diagnosed by specialists for movement disorders according to United Kingdom Parkinson’s Disease Society Brain Bank criteria^34^. To examine the severity of motor symptoms and the disease stage we administered the motor subscale of the Unified Parkinson’s Disease Rating Scale (UPDRS-III) and Hoehn and Yahr staging scale^35^ (See Supplementary Table S2). PD associated dementia was diagnosed according to the presence of a substantial impairment in functions of daily living (Diagnostic and statistical manual of Mental Disorder - DSM-IV, 1995) and the widely accepted clinical criteria for PDD, proposed by the Movement Disorder Society^36^. The diagnosis of mild cognitive impairment was established on clinical consensus criteria, also recently proposed by a task force of the Movement Disorder Society. All MCI patients fulfilled the level II-criteria, which provides a higher diagnostic certainty based on the recommended broad and more detailed cognitive assessment^2^. To diagnose PDD or PD-MCI and rule out cognitive decline in the PD group all patients underwent a thorough neuropsychological evaluation (See Table 1 and Supplementary Table S2) and as depressive symptoms are very common within PDD they we additionally applied the Geriatric Depression Scale (GDS)^37^ (See Supplemental Table S2).

CSF was taken by lumbar puncture mostly between 1 and 4 pm by neurologists. Within 30 min after extraction the sample was centrifuged at 500 g and the supernatant frozen at −80 °C.

### Materials and reagents

NanoPro 1000 reagents were obtained from ProteinSimple (Santa Clara, USA). Dry milk and urea was obtained from Roth (Karlsruhe). 3-[(3-cholamidopropyl)dimethylammonio]-1-propanesulfonate (CHAPS) was from Merck (Darmstadt). DL-Dithiothreitol (DTT), iodoacetamide (IAA) and thiourea was obtained from Sigma (Steinheim, Germany). IPG buffer was from Amersham Biosciences (Freiburg). Sodium dodecyl sulfate was bought from Serva (Heidelberg). Tris pure was obtained from Applichem (Darmstadt). Polyvinylidene fluoride membranes were purchased from Millipore Corporation (Bedford). IPG strips were from GE Healthcare (Munich). The monoclonal mouse anti-human serpinA1 antibody was bought from R&D Systems (MAB1268) (Minneapolis).

### SerpinA1 analysis by 2D-PAGE

For 2D-PAGE analysis 10 μl CSF was prepared with 100 μl lysis buffer (7 M urea, 2 M thiourea, 4% w/v CHAPS, 1% w/v DTT, 1% v/v IPG buffer 3–5.6) for 90 min at room temperature. 2D-PAGE was performed as described in Jesse *et al*.^7^. In brief, proteins were separated on IPG-gel strips pH 3–5.6. For focusing, we used an IPGphor focusing machine (GE Healthcare, Munic, Germany) using an established focusing program^38^.

After equilibration, strips were laid onto gels. Separation was performed with 1x Laemmli buffer (0.025M Tris, 0.192M glycine, 0.1% SDS). Proteins were transferred onto polyvinylidene fluoride membranes. Membranes were blocked and the serpinA1 antibody was applied. After incubation with peroxidase labeled secondary antibody (DAKO, Glostrup, Denmark), blots were developed with ECL (Millipore Corporation, Bedford, Massachusetts) and chemiluminescence was measured with a CCD camera (LAS-1000; Fujifilm, Tokyo, Japan).

### SerpinA1 analysis by capillary isoelectric focusing (CIEF)

After loading, proteins were separated by charge and in the next step immobilized at their isoelectric point by exposure to UV light^39^. The capillary was flowed through with the primary antibody, forming immunocomplexes with serpinA1. After washing the secondary, HRP labeled antibody was added. For detection, chemiluminescent reagents were flowed through the capillary reacting with HRP; the generated light was detected by a CCD camera. Up to 96 capillaries can be processed in one run.

For CIEF analyses native CSF samples were used. Ampholyte G2 premix (pH 4–7) (ProteinSimple) was mixed with pI standards ladder 2 (ProteinSimple) (1 μl/44 μl premix) and vortexed thoroughly. One volume of the sample was mixed with three volumes of premix.

For measurement we used the CIEF platform NanoPro 1000 (ProteinSimple). 10 μl of sample/premix mixture was pipetted into 384 well plates. The detection antibody was diluted 1:100 (v/v) with antibody diluent and 15 μl per well were added. 15 μl secondary antibody (goat anti mouse HRP, Protein Simple) was added. Equal amounts of luminol and peroxide (ProteinSimple) were mixed. 15 μl were loaded per well. Plate was centrifuged for 10 min at 1500 g and 4 C.

The automated assay was programmed in the Compass software (ProteinSimple). The samples were separated by isoelectric focusing for 40 min with 21000 μW. Analytes and standards were subsequently immobilized in a photochemical reaction, followed by two washing steps. The detection antibody was loaded and incubated for 120 min. After two washing steps, luminol/peroxide was loaded for 2 s and chemiluminescence signals were detected^40^.

PI values of the analytes were calculated within Compass software using fluorescent pH standards in each capillary. Chemiluminescence signals were automatically plotted against the pH gradient. For peak identification, a minimum signal to noise ratio of 10 was required.

### SerpinA1 CIEF test reproducibility and stability

For intra and inter test-reproducibility PD and PDD samples where measured six times in one run. The average coefficient of variation (CV) for intra and inter test-reproducibility was calculated as 9.6% and 14.1%, respectively.

For stability analysis samples were 4 times frozen and thawed. No reduction in signal intensity could be found after the freeze and thaw cycles.

### Alpha-synuclein, Tau and Abeta 1–42 measurement

For the measurement of alpha-synuclein, tau and Abeta 1–42 in CSF and serum (only alpha-synuclein) an enzyme-linked immunosorbent assay (ELISA) was used. The ELISA kits were provided by Covance (Alpha-synuclein) and Fujirebio (Tau and Abeta 1–42). For the analysis the manufacturer’s instructions were followed.

### Statistical analysis

Analyses of the peak areas were performed with GraphPad Prism (Version 5.00, GraphPad software Inc) using the One-way Anova test. As a post hoc test Tukey’s Multiple Comparison test was chosen. Results were considered statistically significant when p < 0.05.

## Additional Information

**How to cite this article**: Halbgebauer, S. *et al*. Modified serpinA1 as risk marker for Parkinson’s disease dementia: Analysis of baseline data. *Sci. Rep*. **6**, 26145; doi: 10.1038/srep26145 (2016).

## Supplementary Material

Supplementary Information

## Figures and Tables

**Table 1:** Relevant parameters of all groups.

	CON	PD	PD-MCI	PDD
N	36	22	15	29
Gender m/f	19/17	16/6	8/7	17/12
Age^a^	60 ± 12	69 ± 8	65 ± 9	74 ± 8
Tau (pg/ml)	327 ± 185	256 ± 82	285 ± 109	317 ± 198
Aß 1–42 (pg/ml)	841 ± 295	682 ± 400	977 ± 315	774 ± 262
CSF alpha- synuclein (pg/ml)	1401 ± 689	1037 ± 381	1361 ± 487	1306 ± 512
Serum alpha-synuclein (ng/ml)	29.7 ± 32.9	19.4 ± 21.7	32.6 ± 46.3	17.1 ± 33.9
MMSE^b^	N/A	29 ± 2	28 ± 2	22 ± 5

Tau and Aβ 42 was measured in CSF. Data are indicated as mean ± SD. CON, control; PD, Parkinson’s disease; PDD, Parkinson’s disease with dementia; ^a^Significant difference between PDD and control as well as PDD and PD-MCI patients (p < 0.05); ^b^Significant difference between PDD and PD as well as PDD and PD-MCI patients (p < 0.01).

## References

[b1] de Lau L. M. & Breteler M. M. Epidemiology of Parkinson’s disease. Lancet Neurol 5, 525–535, doi: 10.1016/S1474-4422(06)70471-9 (2006). 16713924

[b2] Litvan I. . Diagnostic criteria for mild cognitive impairment in Parkinson’s disease: Movement Disorder Society Task Force guidelines. Mov Disord 27, 349–356, doi: 10.1002/mds.24893 (2012). 22275317 PMC3641655

[b3] Aarsland D., Andersen K., Larsen J. P., Lolk A. & Kragh-Sorensen P. Prevalence and characteristics of dementia in Parkinson disease: an 8-year prospective study. Arch Neurol 60, 387–392 (2003). 12633150 10.1001/archneur.60.3.387

[b4] Chaudhuri K. R. & Schapira A. H. Non-motor symptoms of Parkinson’s disease: dopaminergic pathophysiology and treatment. Lancet Neurol 8, 464–474, doi: 10.1016/S1474-4422(09)70068-7 (2009). 19375664

[b5] Rongve A. & Aarsland D. Management of Parkinson’s disease dementia : practical considerations. Drugs Aging 23, 807–822 (2006). 17067184 10.2165/00002512-200623100-00004

[b6] Louis E. D., Marder K., Cote L., Tang M. & Mayeux R. Mortality from Parkinson disease. Arch Neurol 54, 260–264 (1997). 9074394 10.1001/archneur.1997.00550150024011

[b7] Jesse S. . Differential sialylation of serpin A1 in the early diagnosis of Parkinson’s disease dementia. PLoS One 7, e48783, doi: 10.1371/journal.pone.0048783 (2012). 23144969 PMC3493604

[b8] Gooptu B. & Lomas D. A. Polymers and inflammation: disease mechanisms of the serpinopathies. J Exp Med 205, 1529–1534, doi: 10.1084/jem.20072080 (2008). 18591408 PMC2442629

[b9] Gettins P. G. Serpin structure, mechanism, and function. Chem Rev 102, 4751–4804 (2002). 12475206 10.1021/cr010170+

[b10] Truong D. D. & Wolters E. C. Recognition and management of Parkinson’s disease during the premotor (prodromal) phase. Expert Rev Neurother 9, 847–857, doi: 10.1586/ern.09.50 (2009). 19496688

[b11] Foltynie T., Brayne C. E., Robbins T. W. & Barker R. A. The cognitive ability of an incident cohort of Parkinson’s patients in the UK. The CamPaIGN study. Brain 127, 550–560, doi: 10.1093/brain/awh067 (2004). 14691062

[b12] Caballol N., Marti M. J. & Tolosa E. Cognitive dysfunction and dementia in Parkinson disease. Mov Disord 22, Suppl 17, S358–366, doi: 10.1002/mds.21677 (2007). 18175397

[b13] Aarsland D., Bronnick K., Larsen J. P., Tysnes O. B. & Alves G. Cognitive impairment in incident, untreated Parkinson disease: the Norwegian ParkWest study. Neurology 72, 1121–1126, doi: 10.1212/01.wnl.0000338632.00552.cb (2009). 19020293

[b14] Hely M. A., Reid W. G., Adena M. A., Halliday G. M. & Morris J. G. The Sydney multicenter study of Parkinson’s disease: the inevitability of dementia at 20 years. Mov Disord 23, 837–844, doi: 10.1002/mds.21956 (2008). 18307261

[b15] Ohrfelt A. . Cerebrospinal fluid alpha-synuclein in neurodegenerative disorders-a marker of synapse loss? Neurosci Lett 450, 332–335, doi: 10.1016/j.neulet.2008.11.015 (2009). 19022350

[b16] Spies P. E., Melis R. J., Sjogren M. J., Rikkert M. G. & Verbeek M. M. Cerebrospinal fluid alpha-synuclein does not discriminate between dementia disorders. J Alzheimers Dis 16, 363–369, doi: 10.3233/JAD-2009-0955 (2009). 19221426

[b17] Reesink F. E. . CSF alpha-synuclein does not discriminate dementia with Lewy bodies from Alzheimer’s disease. J Alzheimers Dis 22, 87–95, doi: 10.3233/JAD-2010-100186 (2010). 20847452

[b18] Parnetti L. . Cerebrospinal fluid Tau/alpha-synuclein ratio in Parkinson’s disease and degenerative dementias. Mov Disord 26, 1428–1435, doi: 10.1002/mds.23670 (2011). 21469206

[b19] Mollenhauer B. . alpha-Synuclein and tau concentrations in cerebrospinal fluid of patients presenting with parkinsonism: a cohort study. Lancet Neurol 10, 230–240, doi: 10.1016/S1474-4422(11)70014-X (2011). 21317042

[b20] Mollenhauer B. . Direct quantification of CSF alpha-synuclein by ELISA and first cross-sectional study in patients with neurodegeneration. Exp Neurol 213, 315–325, doi: 10.1016/j.expneurol.2008.06.004 (2008). 18625222

[b21] Tokuda T. . Decreased alpha-synuclein in cerebrospinal fluid of aged individuals and subjects with Parkinson’s disease. Biochem Biophys Res Commun 349, 162–166, doi: 10.1016/j.bbrc.2006.08.024 (2006). 16930553

[b22] Yamasaki M., Li W., Johnson D. J. & Huntington J. A. Crystal structure of a stable dimer reveals the molecular basis of serpin polymerization. Nature 455, 1255–1258, doi: 10.1038/nature07394 (2008). 18923394

[b23] Devlin G. L., Chow M. K., Howlett G. J. & Bottomley S. P. Acid Denaturation of alpha1-antitrypsin: characterization of a novel mechanism of serpin polymerization. J Mol Biol 324, 859–870 (2002). 12460583 10.1016/s0022-2836(02)01088-4

[b24] Kok K. F. . Heterozygous alpha-I antitrypsin deficiency as a co-factor in the development of chronic liver disease: a review. Neth J Med 65, 160–166 (2007). 17519511

[b25] Osterwalder T., Contartese J., Stoeckli E. T., Kuhn T. B. & Sonderegger P. Neuroserpin, an axonally secreted serine protease inhibitor. EMBO J 15, 2944–2953 (1996). 8670795 PMC450235

[b26] Davis R. L. . Familial encephalopathy with neuroserpin inclusion bodies. Am J Pathol 155, 1901–1913, doi: 10.1016/S0002-9440(10)65510-1 (1999). 10595921 PMC3277299

[b27] Nielsen H. M. . Plasma and CSF serpins in Alzheimer disease and dementia with Lewy bodies. Neurology 69, 1569–1579, doi: 10.1212/01.wnl.0000271077.82508.a0 (2007). 17761554

[b28] Wang Y. . Phosphorylated alpha-synuclein in Parkinson’s disease. Sci Transl Med 4, 121ra120, doi: 10.1126/scitranslmed.3002566 (2012). PMC3302662 22344688

[b29] Liu F., Iqbal K., Grundke-Iqbal I. & Gong C. X. Involvement of aberrant glycosylation in phosphorylation of tau by cdk5 and GSK-3beta. FEBS Lett 530, 209–214 (2002). 12387894 10.1016/s0014-5793(02)03487-7

[b30] Hwang H. . Glycoproteomics in neurodegenerative diseases. Mass Spectrom Rev 29, 79–125, doi: 10.1002/mas.20221 (2010). 19358229 PMC2799547

[b31] Liu F. . Role of glycosylation in hyperphosphorylation of tau in Alzheimer’s disease. FEBS Lett 512, 101–106 (2002). 11852060 10.1016/s0014-5793(02)02228-7

[b32] Yen H. Y. . Effect of sialylation on EGFR phosphorylation and resistance to tyrosine kinase inhibition. Proc Natl Acad Sci USA 112, 6955–6960, doi: 10.1073/pnas.1507329112 (2015). 25971727 PMC4460513

[b33] Liu Y. C. . Sialylation and fucosylation of epidermal growth factor receptor suppress its dimerization and activation in lung cancer cells. Proc Natl Acad Sci USA 108, 11332–11337, doi: 10.1073/pnas.1107385108 (2011). 21709263 PMC3136320

[b34] Hughes A. J., Daniel S. E., Kilford L. & Lees A. J. Accuracy of clinical diagnosis of idiopathic Parkinson’s disease: a clinico-pathological study of 100 cases. J Neurol Neurosurg Psychiatry 55, 181–184 (1992). 1564476 10.1136/jnnp.55.3.181 PMC1014720

[b35] Hoehn M. M. & Yahr M. D. Parkinsonism: onset, progression and mortality. Neurology 17, 427–442 (1967). 6067254 10.1212/wnl.17.5.427

[b36] Emre M. . Clinical diagnostic criteria for dementia associated with Parkinson’s disease. Mov Disord 22, 1689–1707, quiz 1837, doi: 10.1002/mds.21507 (2007). 17542011

[b37] Parmelee P. A. & Katz I. R. Geriatric depression scale. J Am Geriatr Soc 38, 1379 (1990). 2254577 10.1111/j.1532-5415.1990.tb03461.x

[b38] Brechlin P. . Cerebrospinal fluid-optimized two-dimensional difference gel electrophoresis (2-D DIGE) facilitates the differential diagnosis of Creutzfeldt-Jakob disease. Proteomics 8, 4357–4366, doi: 10.1002/pmic.200800375 (2008). 18814332

[b39] O’Neill R. A. . Isoelectric focusing technology quantifies protein signaling in 25 cells. Proc Natl Acad Sci USA 103, 16153–16158, doi: 10.1073/pnas.0607973103 (2006). 17053065 PMC1618307

[b40] Halbgebauer S. . Capillary isoelectric focusing immunoassay as a new nanoscale approach for the detection of oligoclonal bands. Electrophoresis 36, 355–362, doi: 10.1002/elps.201400339 (2015). 25348366

