# Supplementary material for: Modified serpinA1 as risk marker for Parkinson’s disease dementia: Analysis of baseline data
Source: Sci Rep. 2016 May 17;6:26145. doi: 10.1038/srep26145 (PMC4868992; doi:10.1038/srep26145)
Supplement: Supplementary Information [file srep26145-s1.pdf]

Supplementary information

Modified serpinA1 as risk marker for Parkinson´s disease dementia: Analysis of baseline data

Steffen Halbgebauer (1)[#], Magdalena Nagl (1)[#], Hans Klafki (2, 3), Ute Haußmann (2), Petra Steinacker (1), Patrick Oeckl (1), Jan Kassubek (1), Elmar Pinkhardt (1), Albert C. Ludolph (1), Hilkka Soininen (4), Sanna-Kaisa Herukka (4), Jens Wiltfang (3), Markus Otto (1)*

[#] These authors contributed equally to the manuscript

(1) Department of Neurology, University of Ulm, Germany

(2) LVR-Klinikum Essen, Department of Psychiatry and Psychotherapy, Faculty of Medicine, University of Duisburg-Essen, Germany

(3) Department of Psychiatry and Psychotherapy, University Medical Center (UMG), Georg-August-University, Göttingen, Germany

(4) Department of Neurology, University of Eastern Finland and Kuopio University Hospital, Kuopio, Finland

Supplementary Figure S1: Number of serpinA1 spots in respective 2D immunoblots

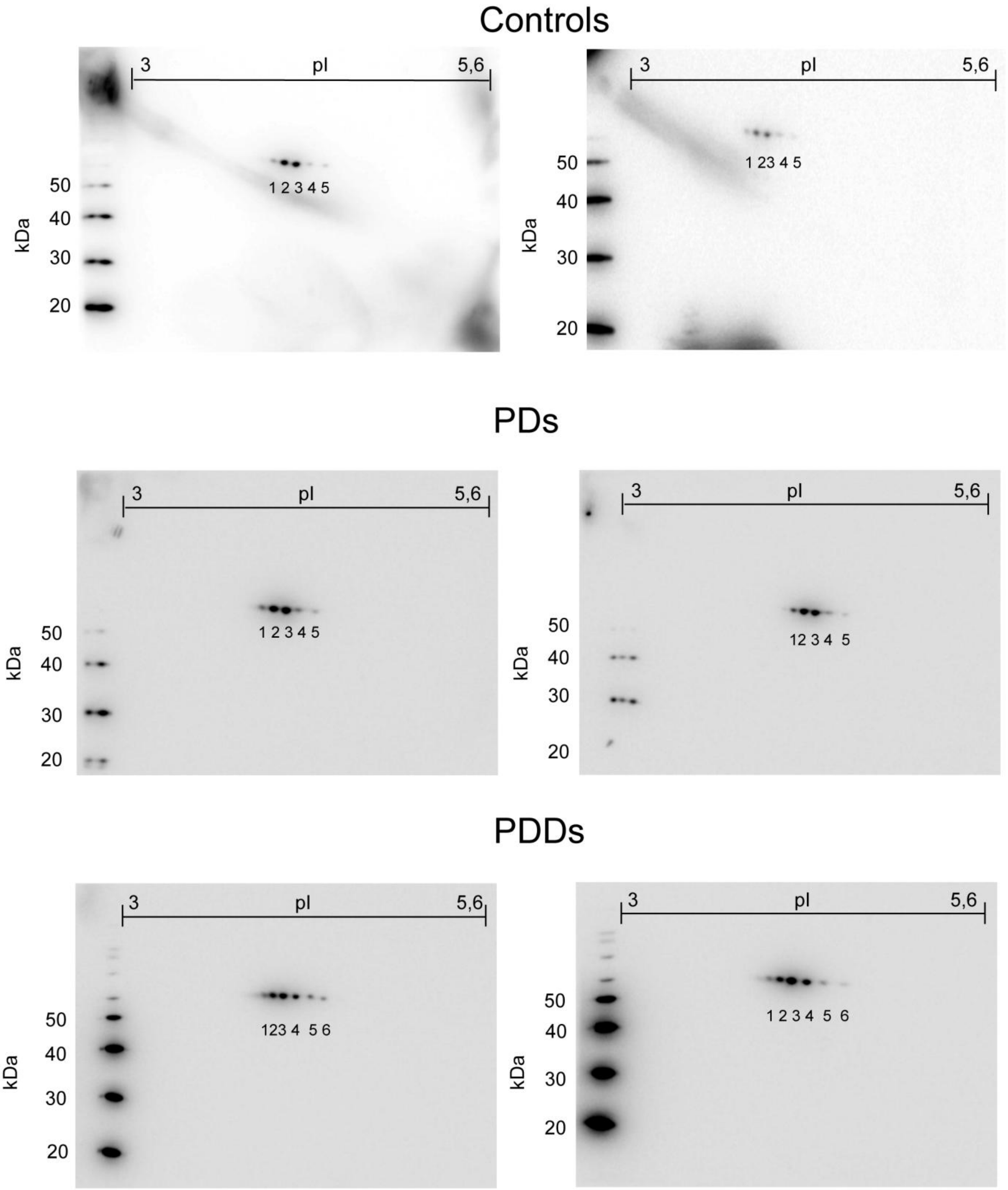

Disease	N	serpinA1 spots	
		≤5	≥6
Control[a]	12	12	0
PD[b]	13	12	1
PDD	12	2	10

Supplementary figure 1: A: Representative 2D serpinA1 immunoblots of controls, PDs and PDDs. In the PDD immunoblots a sixth serpinA1 isoform on the acidic side can be seen.N, Number of samples analyzed , PD, Parkinson's disease; PDD, Parkinson's disease with dementia; a, significant difference between control and PDD ($p<0.001$); b, significant difference between PD and PDD ($p<0.001$).

Supplementary FigureS2: Overall serpinA1 levels of peak 0-6 and Alpha-synuclein quotient (CSF/serum)

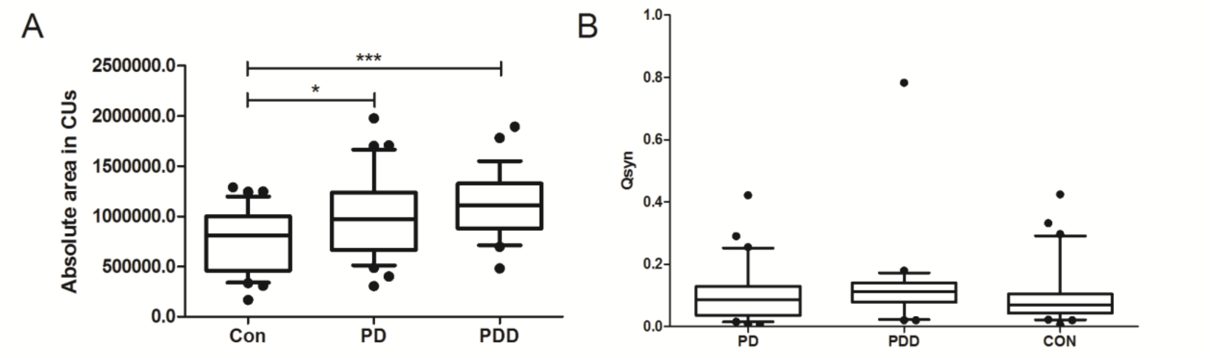

Supplementary figure 2: A; Overall serpinA1 levels of peak 0-6 from 36 control, 37 PD and 29 PDDs. Significant differences between control and PD as well as control and PDD patients. However, no significant difference between PD and PDD patients could be detected. In the box plots the median absolute areas are shown, 25% and 75% percentile, and 10% and 90% whiskers. *, $p<0.05$; ***, $p<0.001$. B; Display of alpha synuclein quotients (CSF/serum) of PD, PDD and control samples. No significant difference between the three groups. In the box plots the median concentrations are shown, 25% and 75% percentile, and 10% and 90% whiskers. CON, control; PD, Parkinson's disease; PDD, Parkinson's disease with dementia.

Supplementary Figure S3: Correlation between age and the absolute area of peak 0

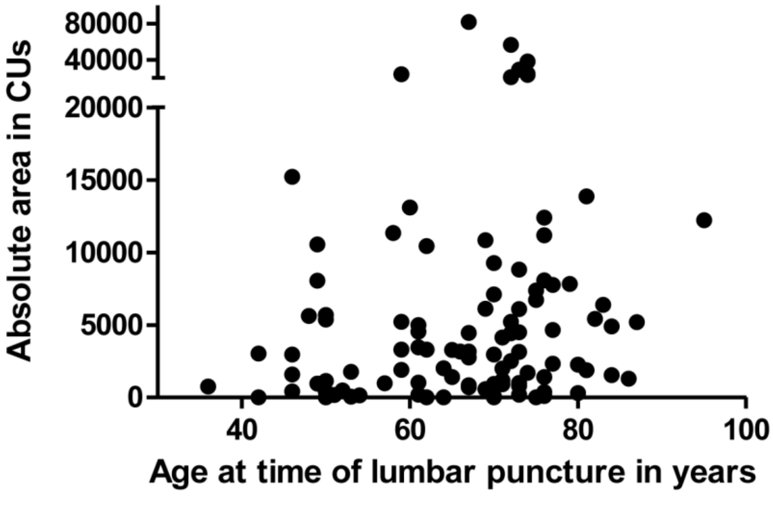

Supplementary figure 3: Correlation between age and the absolute area of peak 0 of all 102 patients. No significant correlation could be detected ($r=0.1275$ and $p=0.2014$).

Supplementary Figure S4: SerpinA1 Peak 0 levels of an additional cohort of 20 non demented control patients

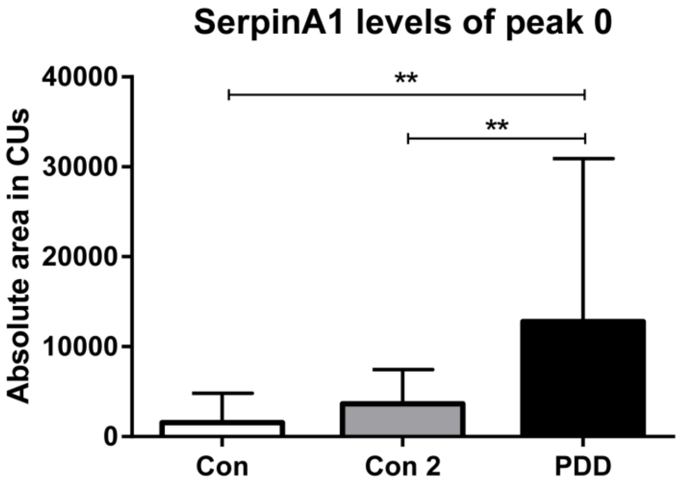

Supplementary figure 4: Peak 0 serpinA1 levels of two control groups and the PDD group. Con, 20 control patients with the mean age of 72.8; Con 2, the 36 control patients included in the manuscript with a mean age of 60; PDD, 29 Parkinson's disease dementia patients (74 ± 8); Error bars indicate SD; ** $p < 0.01$.

Supplementary Table S1: Antiparkinson medication

	PD	PD-MCI	PDD
Treatment with Levodopa (%)	43	40	74
Treatment with dopamine agonists (%)	79	87	63
Treatment with MAO-B inhibitors (%)	21	20	42
Treatment with COMT inhibitors (%)	7	0	5
Treatment with NMDA receptor antagonists (%)	0	0	0
Treatment with anticholinergics (%)	0	13	10
Treatment with Carbidopa/levodopa/entacapone combination (%)	50	13	37

Current administered antiparkinson drugs, expressed as percentage per each patient group. MAO-B, Monoamine oxidase B; COMT, Catechol-O-Methyltransferase; NMDA, N-Methyl-D-Aspartat; PD, Parkinson's disease; PD-MCI, Parkinson's disease with mild cognitive impairment; PDD, Parkinson's disease with dementia;

Supplementary Table S2: Disease duration, clinical and cognitive assessment

		PD	PD-MCI	PDD
**Disease duration[a]**		8±6	3±2	11±5
**Hoehn and Yahr**		2.5±1	2.5±1	3±1
**UPDRS III[a]**		20±9	20±8	32±13
**GDS**		4±4	4±3	5±3
Cognitive Domain	***Assessment***			
Dementia severity rating	CDR[c]*	0±0	0.5±0.5	5±4
Cognitive Screening	PANDA[**1]	25±3	22±4	12±5
Executive functions / Conceptual memory	Semantic fluency[c, †2]	20±5	19±4	14±4
Confrontation Naming / Language functions	Boston Naming[c,†2]	15±0	14±1	12±2
Episodic verbal memory - encoding	Word List Learning[c, †2]	22±3	19±5	13±4
Episodic verbal memory - retrieval	Wordlist Recall[c]	7±2	6±2	3±3
Episodic verbal memory - encoding	Savings Wordlist (%)[b, †2]	83±18	90±36	53±43
Episodic verbal memory - storage	Discriminability (%)[c, †2]	99±9	98±21	89±37
Visuospatial / constructive abilities	Figure Drawing[c,†2]	11±0	10±1	8±2
Episodic nonverbal memory - retrieval	Figure Recall[c, †2]	11±1	9±2	4±4
Episodic nonverbal memory - retrieval	Savings figure (%)[c, †2]	95±8	85±27	44±36
Executive functions	Phonemic fluency[c, †2]	16±5	11±4	8±5
Processing speed / visual scanning	TMT-A (Time)[c,†2]	47±12	64±27	143±40
Mental flexibility / Executive functions	TMT-B (Time)[c,†2]	119±44	170±81	254±71
Verbal short-term / working memory	WMS-R digit span forward / backward[c3]	7±2 / 6±2	6±2 / 5±2	6±2 / 4±1
Spatial short-term / working memory	WMS-R block tapping forward[c]/ backward[c3]	8±1 / 8±2	6±1 / 6±2	4±1 / 3±1

Selective attention / response interference control	Stroop test color-word interference[c4]	32±9	27±10	13±9
Attention / intrinsic alertness	TAP-tonic alertness reaction time (ms)[a5]	312±58	382±96	533±161
Attention / phasic alertness	TAP phasic alertness reaction time (ms)[b5]	304±50	345±72	516±141

PDD patients have a longer disease duration than PD-MCI, but not than PD patients. PDD patients suffer from more severe motor (evaluated with the UPDRS-III motor score, no differences where seen for the rougher but on the other hand more stable Hoehn and Yahr scale) and cognitive symptoms as well as from a greater weakening of every day functioning (CDR) than the non-demented PD-Patients. No group differences were seen for the depression score.Values are expressed as mean ± SD, unless otherwise indicated. Disease duration at time of lumbar puncture. PD, Parkinson's disease; PD-MCI, Parkinson's disease with mild cognitive impairment; PDD, Parkinson's disease with dementia; * sum of boxes score [6]; **contains subtests for episodic and working-memory as well as visuospatial and executive functions; † CERAD-Plus Test battery; [a]*p*<.05; [b]*p*<.01;[c]*p*<.001.

Supplementary material references:

1 Kalbe, E. *et al.* Screening for cognitive deficits in Parkinson's disease with the Parkinson neuropsychometric dementia assessment (PANDA) instrument. *Parkinsonism Relat Disord* **14**, 93-101, doi:10.1016/j.parkreldis.2007.06.008 (2008).

2 Welsh, K. A. *et al.* The Consortium to Establish a Registry for Alzheimer's Disease (CERAD). Part V. A normative study of the neuropsychological battery. *Neurology* **44**, 609-614 (1994).

3 Härting, C., Markowitsch, H.J., Neufeld, H., Calabrese, P., Deisinger, K., Kessler, J. Wechsler Gedächtnis Test — Revidierte Fassung. (2000).

4 Golden, C. J. *A Manual for the Clinical and Experimental Use of the Stroop Color and Word Test*. (Stoelting, 1978).

5 Zimmermann, P., & Fimm, B. in *Applied Neuropsychology of Attention: Theory, Diagnosis and Rehabilitation* (ed In M. Leclercq & P. Zimmermann (Eds.)) 110 - 151 (2002).

6 O'Bryant, S. E. *et al.* Validation of the new interpretive guidelines for the clinical dementia rating scale sum of boxes score in the national Alzheimer's coordinating center database. *Arch Neurol* **67**, 746-749, doi:10.1001/archneurol.2010.115 (2010).
